# Selective electrochemical decomposition of outgrowths and nanopatterning in La_0.7_Sr_0.3_MnO_3_ perovskite thin films

**DOI:** 10.1038/srep07397

**Published:** 2014-12-10

**Authors:** Massimiliano Cavallini, Patrizio Graziosi, Marco Calbucci, Denis Gentili, Raimondo Cecchini, Marianna Barbalinardo, Ilaria Bergenti, Alberto Riminucci, Valentin Dediu

**Affiliations:** 1 Consiglio Nazionale delle Ricerche - Istituto per lo Studio dei Materiali Nanostrutturati (CNR-ISMN), via P. Gobetti 101, 40129 Bologna, Italy

## Abstract

The outgrowth formation in inorganic thin films is a dramatic problem that has limited the technological impact of many techniques and materials. Outgrowths are often themselves part of the films, but are detrimental for vertical junctions since they cause short-circuits or work as defects, compromising the reproducibility and in some cases the operation of the corresponding devices. The problem of outgrowth is particularly relevant in ablation-based methods and in some complex oxides, but is present in a large variety of systems and techniques. Here we propose an efficient local electrochemical method to selectively decompose the outgrowths of conductive oxide thin films by electrochemical decomposition, without altering the properties of the background film. The process is carried out using the same set-up as for local oxidation nanolithography, except for the sign of the voltage bias and it works at the nanoscale both as serial method using a scanning probe and as parallel method using conductive stamps. We demonstrated our process using La_0.7_Sr_0.3_MnO_3_ perovskite as a representative material but in principle it can be extended to many other conductive systems.

Many materials and in particular complex oxides have outstanding properties for several technological applications^1^,^2^,^3^,^4^, but processing them is difficult^5^,^6^. Despite the growth of high-quality thin films, in many areas their technological development is hindered by the formation of a variety of outgrowths, whose size and dispersion are almost uncontrollable^7^.

The outgrowths are themselves part of the films^8^ and, although occasionally they show some structural defects and small variation in their chemical composition^9^, they exhibit the same properties as the background.

Here we propose an efficient method based on local electrochemistry^10^, to selectively decompose the outgrowths of conductive oxides that we named local electrochemical decomposition (LED). By LED we are able to transform conductive outgrowths in insulating objects, therefore preventing short circuit problems in vertical hetero-structures.

As target materials we used La_0.7_Sr_0.3_MnO_3_ (LSMO), a strongly correlated electron system^11^, which is a benchmark material largely used in catalysis^12^ spintronics^13^,^14^,^15^ and solid state memories^16^,^17^ and whose properties are very sensitive to its chemical composition^18^.

LSMO can be electrochemically modified both by oxidation and reduction by anodic or cathodic polarization respectively^12^, in particular it has been proven that the electrochemical reduction acts on the Mn ions, which are reduced from Mn(III) to Mn(II)^12^,^19^ generating of oxygen vacancies in the surface layers of LSMO; this reaction significantly reduces the conductive properties of LSMO surface states^20^.

We demonstrated LED both by scanning probe lithography using, a conductive atomic force microscopy (C-AFM)^21^ and by parallel local electrochemistry, using a stamp instead of a tip^22^,^23^,^24^,^25^. Both approaches are able to confine the electrochemical reaction to the outgrowths, thus allowing their decomposition. Figure 1 shows a schematic drawing of the LED process.

LED occurs in the same configuration as local oxidation, except for the sign of bias voltage: when a conductive tip contacts a surface in a humid ambient (relative humidity (RH) > 50%) a water meniscus condenses between tip and surface forming a nano-electrochemical-cell where tip and substrate are the electrodes (in parallel LED the AFM tip is replaced by a stamp). The water meniscus did not form for RH < 20%. In this case the nano-electrochemical-cell did not form and no reaction was observed (in this condition we only fabricated an ordinary electrical contact between the stamp and the outgrowths). On the other hand, when the meniscus formed, a redox reaction occurred inside the nano-electrochemical-cell upon the application an appropriate bias (oxidation on the positive electrode (anode) and reduction on negative one (cathode)). In particular in LED we used LSMO films as cathode. It must be noted that LSMO, like many oxides, is difficult to be “further oxidised” since the electrochemically active atoms are already in the highest state of oxidation. In this case the application of the traditional local oxidation simply damages of the film (Fig. S1).

Since in LED the oxidation occurs at the tip/stamp electrode, an Au coating prevents the oxidation of the electrode itself (it is the water that is oxidised, due to the higher oxidation potential: Au −1.52 V; H_2_O −1.23 V).

Figure 2 shows the effect of LED on the outgrowths of an LSMO film. Untreated outgrowths exhibit the same electrical conductivity as the background film (Fig 2b). Unexpectedly, by the application of a bias voltage (BV) 6.0 V < BV < 8.0 V (note: this interval is only indicative because it depends on the nature and on the curvature of the tip and on the RH) during the conventional scanning of a C-AFM, only the outgrowths are selectively and irreversibly electrochemically reduced (Fig. 2c). After LED the resistivity of the outgrowths increases by at least three orders of magnitude while no apparent effects were observed in the background (Fig. 2c). The accuracy of this value is limited by the sensitivity of our instrumentation, whose noise level is a few pA, therefore the resistivity of the reduced outgrowths could be much higher. No further effects were observed on outgrowths after repeating the process.

Differently from the case of resistive switching that occurs in LSMO thin films by C-AFM in a dry environment (e.g. when the nanoelectrochemical cell does not form^21^) and on flat zones^26^, in LED the effect on the outgrowths is irreversible and the electrical conductivity cannot be recovered by applying an opposite bias. Given the similarity with the conventional electrochemical reduction^12^ we interpreted our results as a local electrochemical reduction, however we do not exclude some differences related to the application of an electric field, which in the LED set-up is much higher than in conventional electrochemistry.

We attributed preferential reactivity of the outgrowths to the higher electric field generated by the their curvature and to the small alterations in chemical composition or crystalline structural defects^9^.

The evidence that LED is confined to the outgrowths in contact with the tip, prompted us to extend our approach from a serial to parallel, by using a stamp instead of an AFM tip.

For this experiment we used the same set-up developed for parallel local oxidation, using an elastomeric stamp coated with ~30 nm of Au, which allows us to treat areas in excess of 1 × 1 cm^2^ in a single step.

Since the role of the stamp was only to electrically contact the outgrowths, a feature-free, soft stamp could be used for parallel LED.

When the stamp is placed in contact with the substrate it adapts to the sample morphology allowing the electrical contact to be made with all the outgrowths (Fig. 1b). The presence of the outgrowths itself prevents the formation of a water meniscus between the stamp and the background film and, as a consequence, the electrochemical reaction is prevented, regardless of the RH. This configuration allows the application of a high and long BV (>30 V for >30 s) that, though being extreme conditions for conventional local electrochemistry, allow in this case the electrochemical reduction of all the outgrowths in contact with the stamp. No contrast was observed on the treated outgrowths by magnetic force microscopy and surface potential microscopy, while scanning electron microscopy shows a contrast between treated and untreated outgrowths, which is due to different electrical conductivity (Fig. S2 in SI). Figures 2d, 2e show the morphology and the conductive map of the surface of an LSMO particularly rich in outgrowths.

In order to check that LED does not affect the physical properties of the LSMO thin film background, magnetoresistance (MR) measurements were performed on very thin films (7 ± 3 nm), in which a significant impact of the process could be expected due the extremely thin thickness (see details in methods). Figure 3, shows the effect of parallel LED on MR before and after the treatment.

Figure 3a shows that the electrical resistance of the film is indeed affected by the LED. This possibly means that both the outgrowths and the film underneath them are reduced, causing a decrease in the effective cross section of the conductive portion of the film. This increase in resistance is of course an intrinsic consequence of any attempt to eliminate a part of a sample, and should not be considered as a side effect of the proposed technique. More importantly, figure 3b clearly shows that the reduction process did not affect the magneto-resistive properties of the film, demonstrating that the chemical and structural properties of the background film were not altered.

By using C-AFM and by applying bias voltages >8.0 V and/or by repeating scans in the same area, also the background could be electrochemically reduced. In this case we observed also a decrease of the electrical conductivity of the background higher than two orders of magnitude, which is often associated with an increase in roughness (Fig. S3, S4 in SI).

Considering that the morphology of LSMO film consists of grains whose mean diameter is ~20 nm as measured by AFM, we took the grains as the smallest outgrowth, pushing LED toward its resolution limits, viz. the electrochemical reduction of a single grain (i.e. using LED as a conventional scanning probe lithography method). Figure 4 shows an example of an ordered pattern of (anti)dots ~20 nm size produced by applying LED along a square grid, with a 60 nm pitch. In this case the electrochemical reduction occurs at the level of the film background leaving the grains unaltered in between line scans of the C-AFM. The surface results in an ordered array of conductive anti-dots embedded in an insulating matrix.

In summary, we proposed a new and efficient method to address the problem of short circuiting by outgrowths in conductive thin films that are detrimental for many systems of great scientific and technological relevance. The process exploits local electrochemical decomposition and the higher reactivity of outgrowths compared to the background film. Our procedure is single step, cheap, versatile and reproducible, and allows one to transform conductive outgrowths in inert objects without altering the substrate properties.

A peculiarity of our approach is the use electrochemical reduction configuration, which compared to the more common local oxidation, shows clear advantages for oxides that often cannot be “further oxidized”. LED can be downscaled as an scanning probe lithographic technique until the formation of nanometric structures. It can be serial (by C-AFM < 20 nm resolution) or parallel (demonstrated to scale >1 cm × 1 cm).

We gave a demonstration of our process by using LSMO grown by channel spark ablation as a representative material but we can easily extend this process to other conductive systems both using electrochemical reduction and electrochemical oxidation configuration. In this respect, our work represents an important technological advance in view of the application of conductive oxides in electronics and spintronics. This ongoing work will enable the development a variety of systems and techniques, which are now limited by the formation of conductive outgrowths^27^.

## Methods

### Serial local electrochemical decomposition

Serial LED was performed using a commercial conductive AFM (MultiMode 8, Bruker) operating in a controlled atmosphere. Relative humidity (RH < 10% for conduction measurements, and RH > 65% for LED) was obtained by fluxing dry or moist nitrogen, monitoring the RH with a hygrometer. Si-doped, Au-or Pt-coated cantilever AFM tips for contact mode (NT-MDT CSG10, with typical curvature radius of a tip of 10 nm) were used to perform LED. To treat outgrowths a bias voltage of 6.0 V < BV < 8.0 V was applied during the AFM scans in contact mode while applying a loading force ranging from 1 to 5 nN (no effect linked to the loading force was observed in LED). Typically we observed currents ranging from 0.1 to 100 nA, depending from the sample, RH and tip radius.

Conductive and topographic maps where obtained with the same experimental set-up used for LED but with RH > 10%.

The topographic images were corrected line-by-line for background trend effects by removal of the second-order polynomial fitting. Image analysis was carried out using the open source SPM software Gwyddion (www.gwyddion.net).

### Parallel local electrochemical decomposition

Parallel LED was performed using a home-made apparatus as described in ref. 23 in the text. It consists of a press with a conductive stamp, with the substrate fixed on a rigid sample holder on the bottom side. The substrate and the stamp were connected electrically to the voltage source (ELIND Model 3232). The system was inserted inside the sealed chamber (Tupperware, USA) with controlled RH obtained by fluxing dry or moist nitrogen, the exact RH was controlled by a hygrometer (PCE Model 313-A). The RH was kept stable by fluxing moist nitrogen. When the RH reached 90%, the stamp and substrate were put in contact by the downward motion of the micrometric screw. In this configuration, the substrate/ water/ metal stamp system formed an electrochemical cell, with the LSMO substrate acting as the cathode, the water layer between silicon and stamp as the electrolyte and the metal stamp as the anode. Then a bias voltage of 30 V DC with limited current (typically 200 mA) was applied between the electrodes for 30 s.

### Stamps

The elastomeric polydimethylsiloxane (PDMS, Sylgard 184 Down Corning) stamps were prepared by replica moulding of a silicon wafer. The curing process was carried for 1 hour at 70°C. Once cured, the replica was peeled off from the master and washed in pure ethanol to remove uncured polymer. After that, the PDMS replica was coated with a 100 nm thick film of metal by thermal evaporation.

### La_0.7_Sr_0.3_MnO_3_ films growth

Thin films of La_0.7_Sr_0.3_MnO_3_ were grown by channel spark ablation (CSA) on SrTiO_3_ (100) (STO) substrates. STO substrates were cleaned for 15–20′ at in a ultrasonic bath of Extran® (NaOH based solvent), then in three ultrasonic baths in deionized water lasting 10′ each (with fresh deionized water for each bath), then in a 15′ ultrasonic bath in acetone and finally for 15′ in isopropanol (acetone and isopropanol of spectroscopic grade). STO substrates were heated in an oxygen environment (3.5 **·** 10^−2^ mbar with a base vacuum of 4 **·** 10^−4^ mbar) up to 890°C as measured by an optical pyrometer. The ablation of a stoichiometric target with density higher than 95% of the theoretical value was performed by CSA in an oxygen atmosphere around 3.5 **·** 10^−2^ mbar and with an accelerating voltage about 10 kV at a repetition rate of 6 Hz. After the deposition the samples were quenched in vacuum to about 400°C and annealed in vacuum for 10′, then cooled to room temperature. Samples particularly over-rich of outgrowths were selected to demonstrate LED^28^. Typical film thickness used for our work was 7 ± 3 nm as ultra thin films, 25 ± 3 nm to develop the process.

### Magnetotransport measurements

Magnetotransport measurements were performed on 1 mm × 5 mm × 7 ± 3 nm film of LSMO using a two-contact configuration. Contacts on LSMO stripes were made manually by Indium. MR measurements were carried out by using a Keithley 236 Source Measure Unit, applying a −0.1 V bias voltage and by sweeping the magnetic field from −3000 Oe to 3000 Oe. MR values (%) were calculated as [R(H = 0 Oe) – R(H = 800 Oe)]/ R(H = 0 Oe). Parallel LED was applied in between the electrical contacts and it was performed in more than 90% of the total area of the sample.

## Author Contributions

Mas.Cav. and A.R. are responsible for designing and providing guidance for the experiments and for editing and proofreading the paper. P.G., D.G., Mar.Cal., R.C. and M.B. are responsible for all the experiments sample preparation and characterization; I.B. and V.D. contributed to data interpretation and the organization of the experiments. All authors contributed to write the paper.

## Supplementary Material

Supplementary Information Supplementary Info

## Figures and Tables

**Figure 1:**
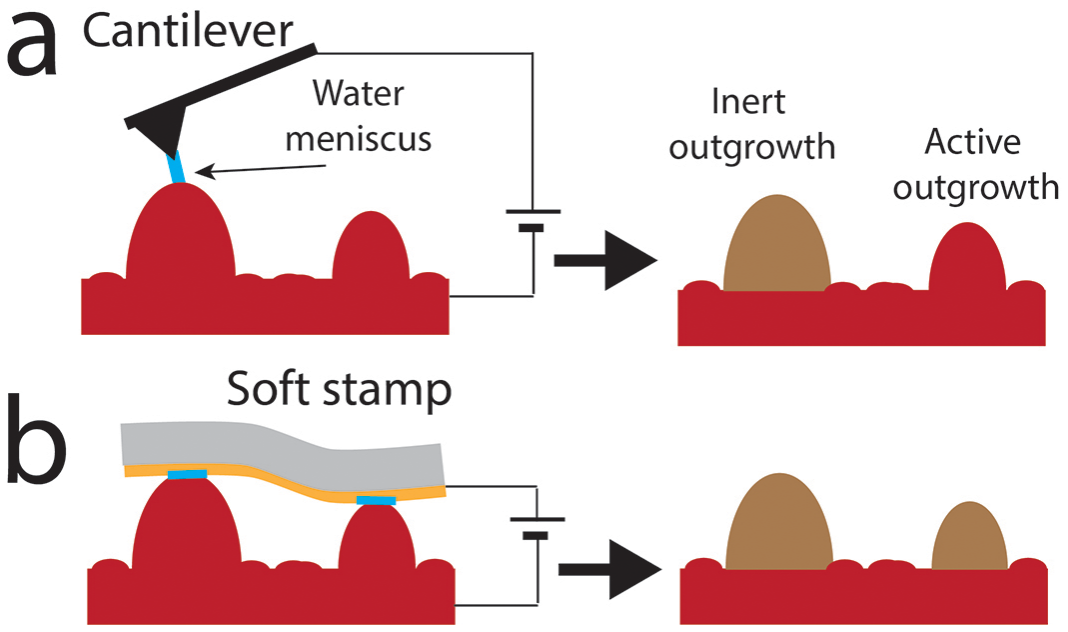
Scheme of local electrochemical decomposition (LED). (a) Serial configuration, using the tip of an atomic force microscope and (b) parallel configuration, using a featureless stamp.

**Figure 2:**
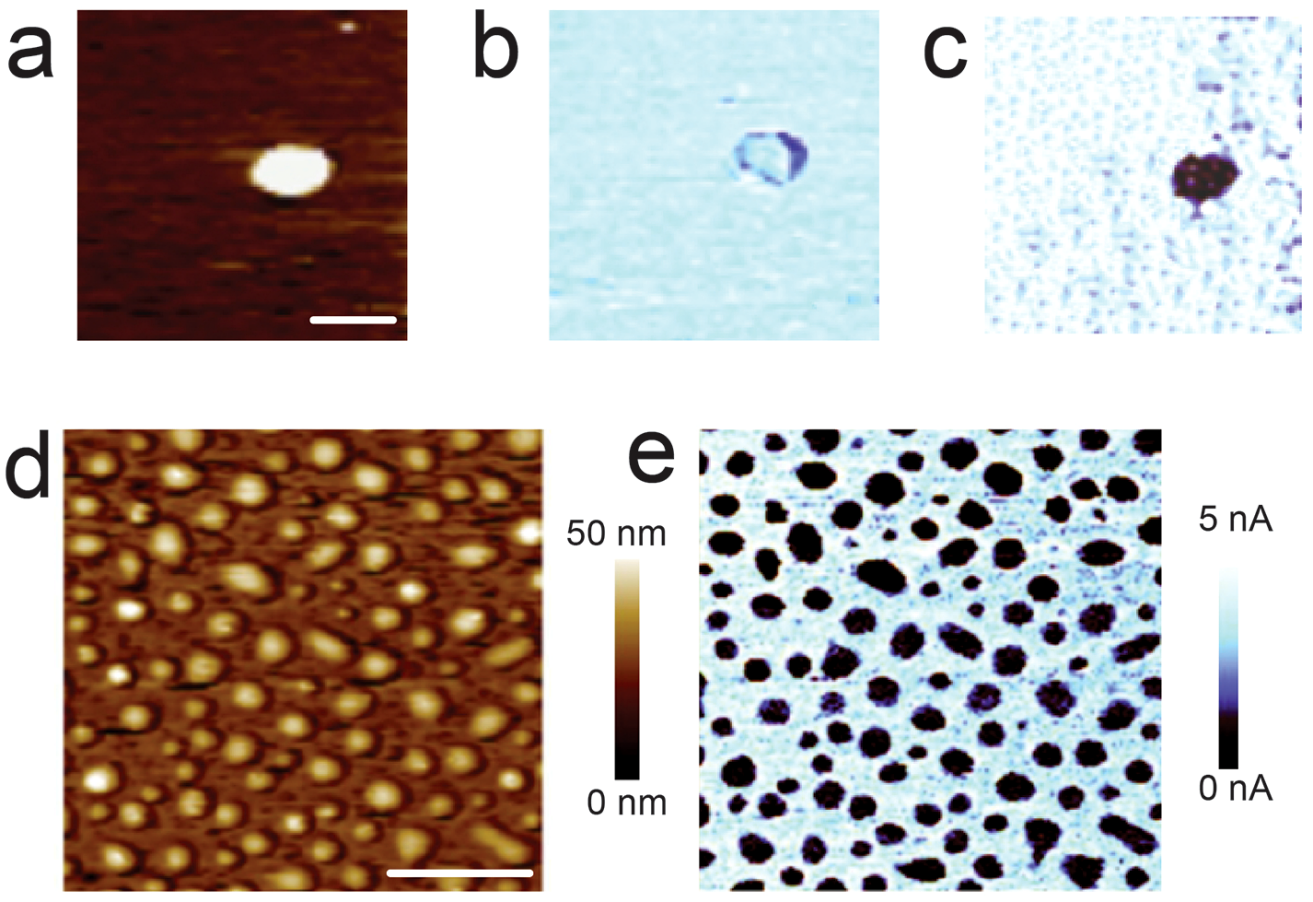
Effect of LED on individual outgrowths of a LSMO thin film grown by channel spark ablation. (a) Topography of an isolated outgrowth; bar 200 nm, Z scale 0–20 nm. (b) Corresponding conductivity map measured at −8.0 V in contact mode in dry nitrogen (RH < 10%); Z scale 0–7 nA. (c) Corresponding conductivity map after local electrochemical reduction performed at RH 65%; Z scale is 0–5 nA. The outgrowth became irreversibly isolating. (d) Morphology of LSMO thin film rich of outgrowths. (e) Corresponding conductive map recorded at −8.0 V.

**Figure 3:**
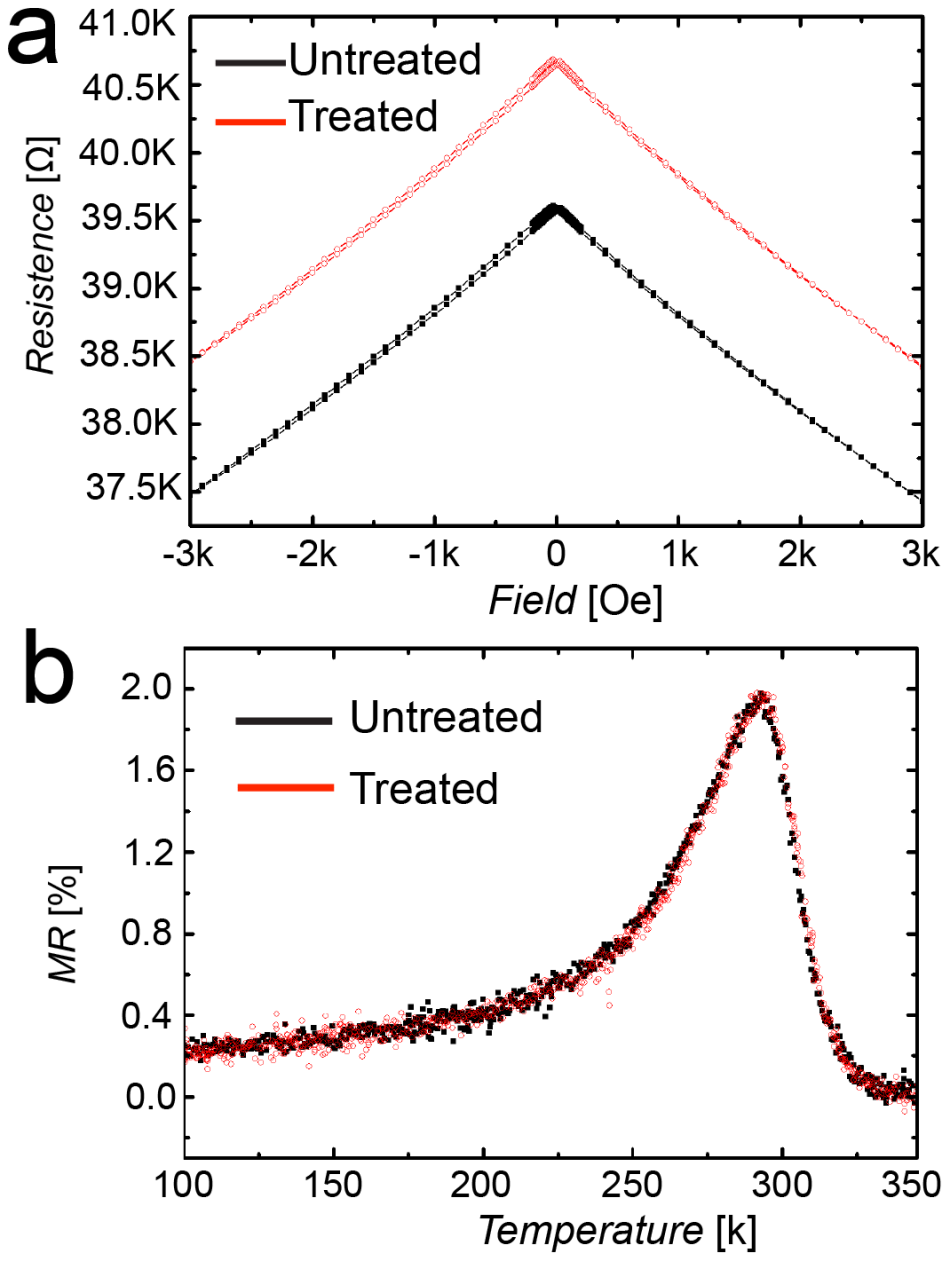
LED effect on magnetoresistance. The curves were recorded on LSMO 10 nm thick film treated by parallel LED. (a) Trend of electrical resistance versus magnetic field recorded at 300 K. (b) Trend of magneto-resistance recorded at 800 Oe as a function of temperature. No significant effect of LED treatment was observed in MR.

**Figure 4:**
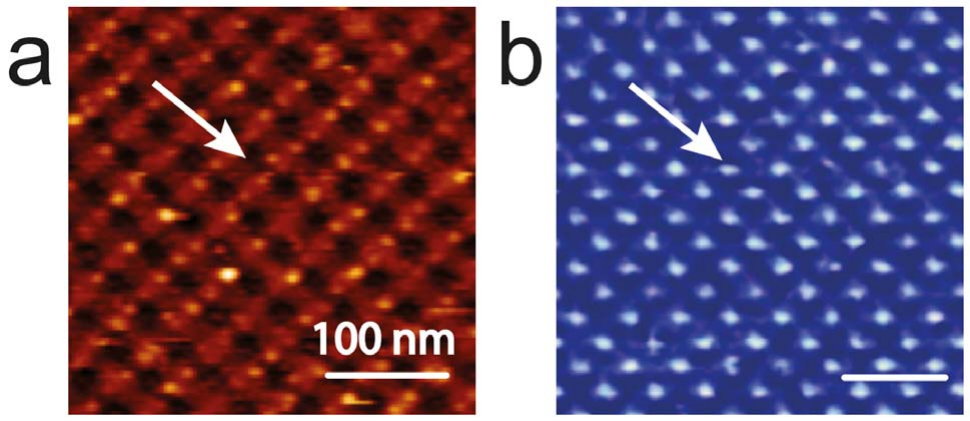
Pattern of LSMO anti-dots 20 nm diameter fabricated by LED. (a) AFM Topography. The arrow indicates an anti-dot, Z scale 0–10 nm. (b) Corresponding electrical conductivity map recorded at 7.0 V bias voltage. Z scale 0–5 nA.
