# Supplementary material for: Selective electrochemical decomposition of outgrowths and nanopatterning in La0.7Sr0.3MnO3 perovskite thin films
Source: Sci Rep. 2014 Dec 10;4:7397. doi: 10.1038/srep07397 (PMC4261174; doi:10.1038/srep07397)
Supplement: Supplementary Information — Supplementary Info [file srep07397-s1.pdf]

Selective electrochemical decomposition of outgrowths and nanopatterning in $La_{0.7}Sr_{0.3}MnO_3$ perovskite thin films

Massimiliano Cavallini[1]*, Patrizio Graziosi[1], Marco Calbucci[1], Denis Gentili[1], Raimondo Cecchini[1], Marianna Barbalinardo[1], Ilaria Bergenti[1], Alberto Riminucci[1]* & Valentin Dediu[1]

1-Consiglio Nazionale delle Ricerche - Istituto per lo Studio dei Materiali Nanostrutturati (CNR-ISMN), via P. Gobetti 101, 40129 Bologna, Italy.

* E-mail: Massimiliano.cavallini@cnr.it

SUPPORTING INFORMATIONS

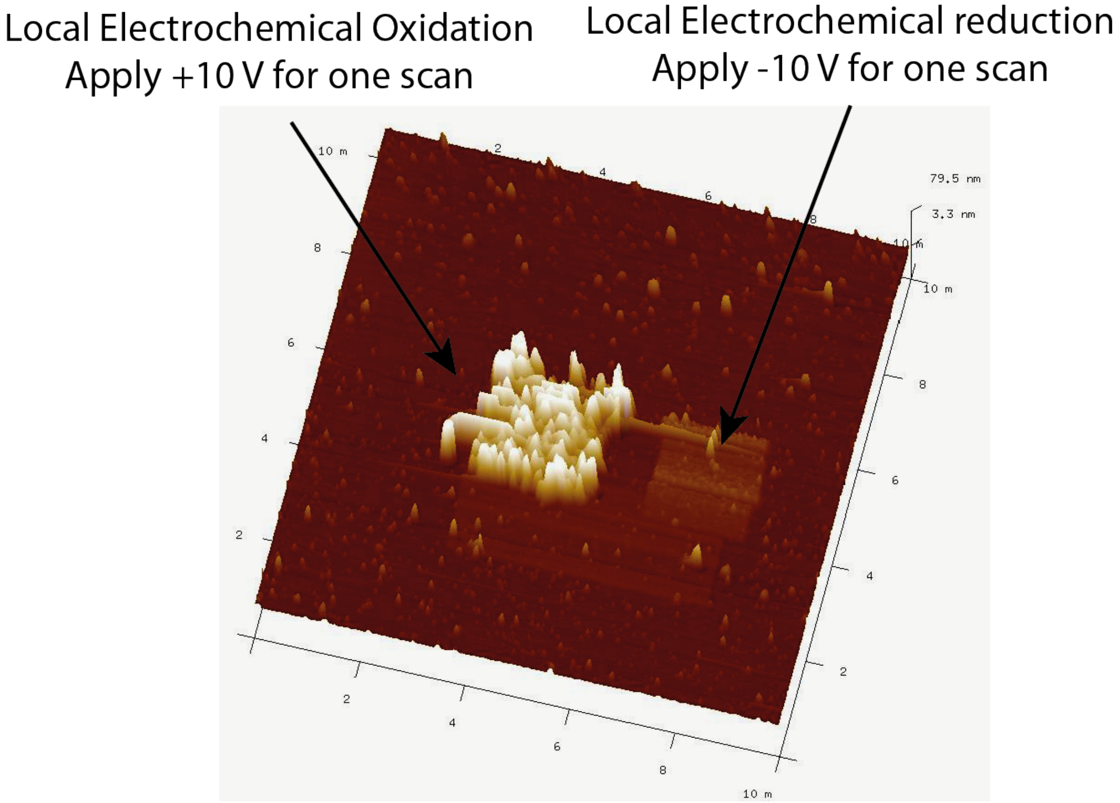

Figure S1. Morphological effect of local electrochemical oxidation compared to LED on $La_{0.7}Sr_{0.3}MnO_3$ perovskite thin films ~40 nm thick. Local electrochemical oxidation damages the film.

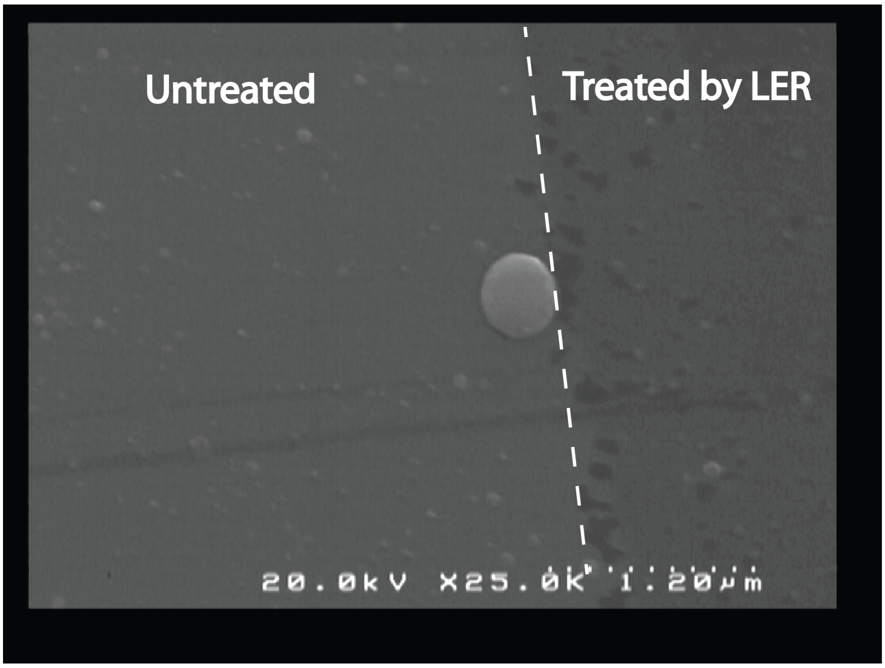

Figure S2. Effect of selective LED of outgrowth in $La_{0.7}Sr_{0.3}MnO_3$ thin films (~20 nm thick) observed by scanning electro microscopy. The sample is fabricated by C-AFM applying a -7.5 V for one scan (RH 65%) in the right part of the image. The dashed line shows the boundaries between treated and untreated zones. The outgrowths on treated zone appear dark because their electrochemical reduction. In order to better show the differences the contrast of the image is graphically enhanced.

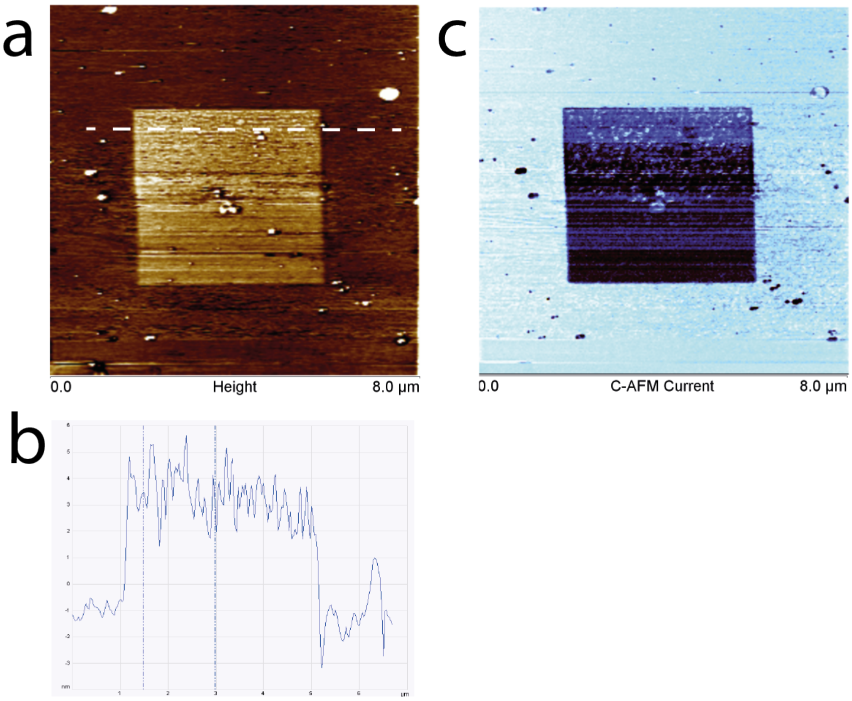

Figure S3. Topographic effects of hard-LED (i.e. applying -10 V with RH 65%) on $La_{0.7}Sr_{0.3}MnO_3$ thin films (~20 nm thick) background. a) Topographic effect (z scale is 0-10 nm), b) Line profile recorded along the white line of a. c) Corresponding conductivity map recorded at -7.0 V in dry atmosphere.

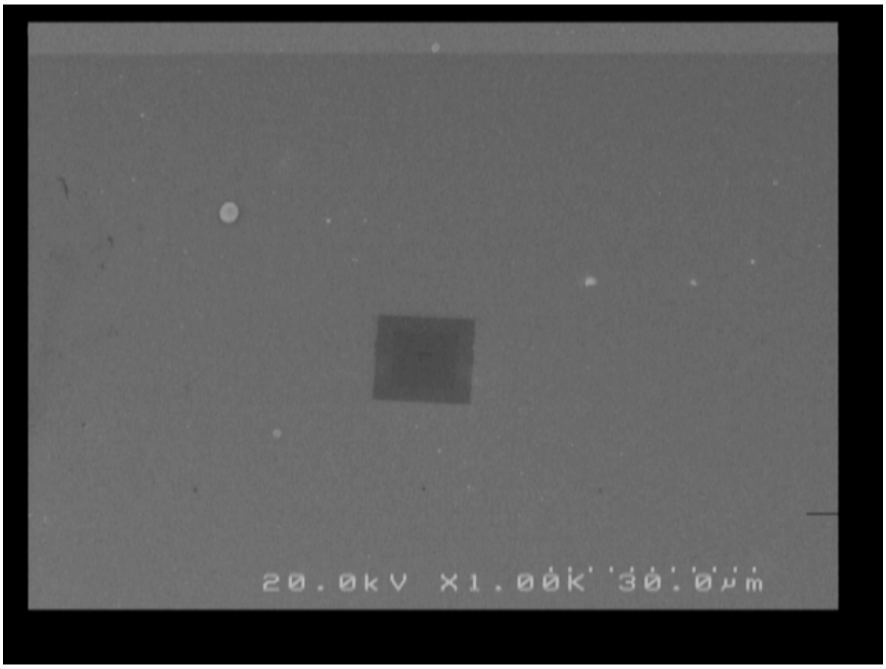

Figure S4. Effect of hard-LED on $La_{0.7}Sr_{0.3}MnO_3$ thin films background observed by scanning electro microscopy. The dark zone at the centre of the image was obtained by LER applying -10 V (RH 65%). The small difference of contrast between the inner part of the treated zone and the external part is due to the number of scans (1 in the outer zone and 2 in the inner one).
